# Supplementary material for: The Perspective of Physical Education Teachers in Spain Regarding Barriers to the Practice of Physical Activity among Immigrant Children and Adolescents: A Qualitative Study
Source: Int J Environ Res Public Health. 2021 May 24;18(11):5598. doi: 10.3390/ijerph18115598 (PMC8197202; doi:10.3390/ijerph18115598)
Supplement: Supplementary file 1 [file ijerph-18-05598-s001.zip › ijerph-1203384-supplementary.pdf]

Transcription process

Full verbatim literal transcription

Researcher's field notes and description

Thematic indutive analysis

Participant's narratives --> Coding process --> Grouping codes into Categories --> Identifying Themes

Example

**Step 1: Identifying Participant's Narratives**	**Step 2: Coding process**	**Step 3: Grouping codes**	**Step 4: Identifying Theme**
"They are divided into subjects that can generate employment and those that can' t, such as physical education." (p17)<br>*"Subjects are not given the same value. Many parents are clear: fail physical education and nothing happens, fail maths and it's the end of the world." (P18)*	Code 1: Subjects that generate employment<br>Code 2: Physical education offers no future<br>Code 3: Parents provide guidance on which subjects are better than others<br>Code 4: All subjects do not have the same value	Category: Prioritization subjects	Future professional expectations
" Parents have a lot to say. If they don't support physical activity, everything becomes more complicated. It' s a path towards a healthy future." (P4)<br>*"There is a bit of everything, kids who think that physical activity is just a hobby, like a game console, and others who use it to escape from problems at home." (13)*	Code 5: Parents influence the value of physical activity<br>Code 6: Pathway to healthy habits<br>Code 7: Physical education has no value, it's just exercise.<br>Code 8: Physical activity as a means of escaping conflict	Category: value to the practice of physical activity	

Figure S1. Example of codification process.
